# Deep PUF: A Highly Reliable DRAM PUF-Based Authentication for IoT Networks Using Deep Convolutional Neural Networks

**DOI:** 10.3390/s21062009

**Published:** 2021-03-12

**Authors:** Fatemeh Najafi, Masoud Kaveh, Diego Martín, Mohammad Reza Mosavi

**Affiliations:** 1 ETSI de Telecomunicación, Universidad Politécnica de Madrid, Av. Complutense 30, 28040 Madrid, Spain; fa_najafi@elec.iust.ac.ir; 2 Department of Electrical Engineering, Iran University of Science and Technology, Tehran 13114-16846, Iran; m_kaveh@elec.iust.ac.ir (M.K.); m_mosavi@iust.ac.ir (M.R.M.)

**Keywords:** DRAM latency-based PUF, IoT, authentication, convolutional neural network

## Abstract

Traditional authentication techniques, such as cryptographic solutions, are vulnerable to various attacks occurring on session keys and data. Physical unclonable functions (PUFs) such as dynamic random access memory (DRAM)-based PUFs are introduced as promising security blocks to enable cryptography and authentication services. However, PUFs are often sensitive to internal and external noises, which cause reliability issues. The requirement of additional robustness and reliability leads to the involvement of error-reduction methods such as error correction codes (ECCs) and pre-selection schemes that cause considerable extra overheads. In this paper, we propose deep PUF: a deep convolutional neural network (CNN)-based scheme using the latency-based DRAM PUFs without the need for any additional error correction technique. The proposed framework provides a higher number of challenge-response pairs (CRPs) by eliminating the pre-selection and filtering mechanisms. The entire complexity of device identification is moved to the server side that enables the authentication of resource-constrained nodes. The experimental results from a 1Gb DDR3 show that the responses under varying conditions can be classified with at least a 94.9% accuracy rate by using CNN. After applying the proposed authentication steps to the classification results, we show that the probability of identification error can be drastically reduced, which leads to a highly reliable authentication.

## 1. Introduction

A large number of modern cryptographic protocols are based on physical unclonable functions (PUF) implementations, which are used for key agreement and device authentication [1,2,3,4,5]. Memory-based PUFs are popular among other implemented PUFs due to being a major component in many electronic devices and requiring minimum (or no) additional circuit for PUF operation [6,7]. Dynamic random access memory (DRAM)-based PUFs provide large address space and utilize several controllable properties to generate unique identifiers for identification and authentication purposes [8,9,10,11]. Recently, researchers have proposed DRAM latency-based PUFs, which can be used to provide random device signatures by exploiting the timing parameters (e.g., activation (t_RCD_), precharge time (t_RP_), etc. [12,13]). Reliability and robustness are two fundamental properties of a desirable PUF, which prove the independence of output responses on internal/external noises and ambient conditions. Most of the existing PUFs use some post-processing techniques, which require helper data algorithms and complex error correction codes (ECCs) to extract reliable responses and conduct a proper authentication procedure [8,14]. However, these methods cause significant hardware/computational overheads and additional Non-volatile memory (NVM) to store helper data in addition to their security defects [15,16,17]. Most of the proposed DRAM PUFs employ different pre-selection mechanisms to eliminate dependent or unstable cells and decrease the ECC overheads [12,13]. Pre-selection mechanisms consist of running multiple tests on the PUF and selecting the qualified cells using selection algorithms. These approaches limit the challenge-response pairs (CRP) space and entail additional runtime and costs. In such a way, resource-constrained nodes in the internet of things (IoT) applications cannot benefit from a DRAM PUF-based authentication.

In [18], Yue et al. proposed a DRAM-based authentication scheme without the need for PUFs in which a deep convolutional neural network (CNN) is utilized to authenticate DRAM Integrated Circuits (ICs) using raw power-up values. This method is secure against machine learning attacks and eliminates error correction mechanisms. However, this scheme can result in increased runtime because of requiring power cycles to extract power-up characteristics. Using CNN to extract unique features of DRAM at the device side can cause extra overhead for resource-constrained nodes.

Most of the existing PUF-based authentication methods for IoT applications exploit strong PUFs, such as delay-based PUFs, which require dedicated circuits. Additionally, most of these methods rely on complex error correction algorithms to extract reliable responses, leading to add extra overheads to the device [19,20]. Using DRAMs as the major components of many devices can eliminate the need for dedicated PUF circuits. Some latency-based DRAM PUFs, which do not need power cycles and waiting periods, can be evaluated as appropriate approaches to configure a lightweight authentication [12,13,18,21,22]. In this paper, we propose deep PUF that utilizes all latency failures in different DRAM blocks to expand the CRP space and organize a robust authentication without employing any pre-selection algorithms or post-correction mechanisms. We also move the entire burden of CNN implementation and device identification to the server-side to overcome the challenges of resource-constrained devices. The major contributions of this paper can be summarized as follows:

- We propose deep PUF as a two-stage mechanism, including multi-label classification and challenge verification, to provide a robust and lightweight device authentication without error-correcting codes and other pre-filtering methods.
- We implement two types of latency-based proposals (t_RCD_ and t_RP_ PUFs) as the fast runtime accessible DRAM PUFs and analyze their characteristics to train the CNN.
- Finally, we develop a CNN model using experimental data and analyze the robustness and security of the proposed deep PUF.

The remainder of this paper is organized as follows: Section 2 explains the background and motivations. Section 3 presents the proposed deep PUF. Section 4 and Section 5 demonstrate DRAM experiments and CNN development results, respectively. Section 6 discusses deep PUF performance and security, and Section 7 concludes the paper and mentions future directions.

## 2. Background and Motivation

### 2.1. DRAM Operation and Timing Parameters

**Figure 1:**
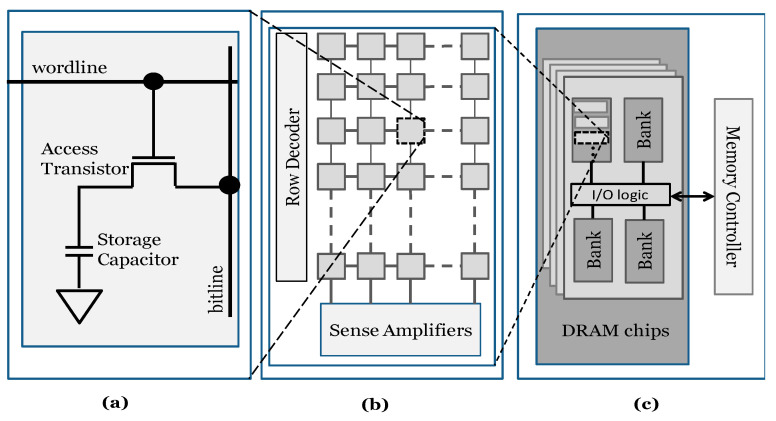
DRAM organization [12]. (**a**) DRAM cell. (**b**) DRAM subarray. (**c**) DRAM device.

**Figure 2:**
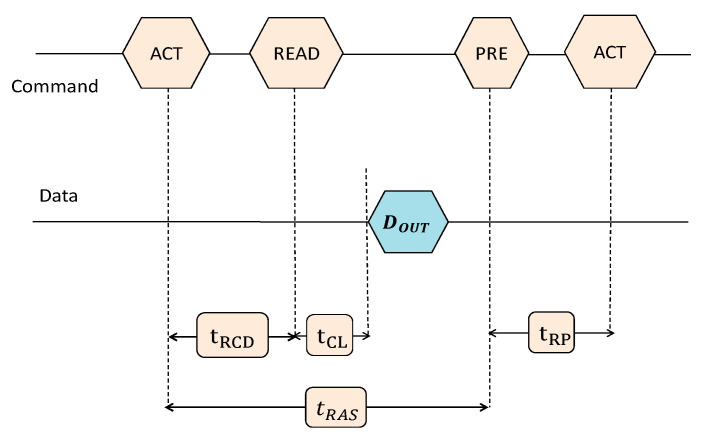
DRAM timing at read operation [13].

The hierarchy of a DRAM device organization is presented in Figure 1. As shown in Figure 1a, a DRAM cell is the lowest level of DRAM structure, which stores one bit of data based on the charge of its capacitor. A cell encodes value “1” when the capacitor is fully charged and value “0” as it is fully discharged. The cells are written to or read from using the access transistors, which are enabled by the *wordline* and the *bitline* connects the cells of each column. Figure 1b shows how these components form a two-dimensional subarray of a DRAM module. The combination of numerous subarrays forms a bank and banks are cooperated to organize a DRAM chip, as shown in Figure 1c. To activate a row, the row decoder enables the corresponding *wordline,* and then the stored information is transferred to the sense amplifiers. When the row is accessed, the data can be read/written using *rd/wr* commands [23]. Each read operation consists of multiple states, and the memory controller produces different commands and manages the states (see Figure 2). In the precharge state, all *bitline*s are precharged, and other open *wordline*s are deactivated after using the precharge (PRE) command. Before any *rd/wr* command can be generated, the corresponding *wordline* must be opened via the activation (ACT) command. Next, *rd/wr* commands can be sent to the opened row, subject to a minimum required time (activation time, t_RCD_). For a subsequent read/write operation, it is necessary to issue a PRE command to deactivate the opened row. The next row will be accessible after a specified time named precharge time (t_RP_). There are also other timing parameters such as t_RAS_ and t_CL_ that are used by the memory controller to manage DRAM operations [24,25,26,27].

### 2.2. DRAM PUF Technologies

In this section, we explain the existing DRAM-based PUFs and their strategies to enhance robustness and reliability.

A variety of works have discussed the concept of using DRAM as the major hardware of most modern systems to organize an intrinsic PUF. DRAM cells do not have zero values at the start-up time and the capacitor in each cell is initialized to a random value caused by manufacturing variations, which is utilized to provide device signatures and configure a DRAM PUF [22]. The requirement of power cycles and a period of time before response generation to extract unbiased signatures are the most important challenges of this technique. Retention-based PUF is another well-studied mechanism, which generates DRAM PUF-based random and unique patterns by preventing the refresh operation for a period of time (waiting time). Retention-based PUFs require a long period of time to extract sufficient failure. This method exploits the pre-selection of blocks and helper data algorithms to extract the robust responses for key generation and authentication purposes. Therefore, retention-based PUFs include significant time, hardware and storage overheads.

#### Latency-Based DRAM PUFs

As mentioned in the DRAM organization, there are specified timing constraints to schedule the DRAM operations correctly. Altering these parameters can affect the reliability of DRAM information and result in data leakage. Latency-based structures benefit this feature to construct a PUF. The t_RCD_-based PUF is formed by reducing the minimum time period required to activate rows to be accessed [12]. This structure applies a filtering mechanism to eliminate unstable bits in different iterations to enhance the PUF’s robustness and repeatability. A separate DRAM rank is needed to count and store the latency failures of each iteration. The evaluation time of PUF responses is noticeably increased due to the filtering phase. However, this mechanism is not adequate, and ECC approaches are still required to perform a reliable PUF.

Another technique is proposed in [13] that is based on t_RP_-reduction and disrupts precharge procedure to obtain erroneous data. The t_RP_-based technique categorizes the cells on the basis of their dependency on input patterns and measurements. Then, only the independent cells are qualified to be used. Next, the specific selection algorithm is designed to choose the acceptable cells and improve the robustness of PUF. In such a scenario, the CRP space is noticeably contracted, and the effects of environmental variations are not considered.

### 2.3. Post-Processing and Pre-Selection Algorithms

Most of the existing PUF technologies such as DRAM PUFs utilize helper data algorithms and ECCs to improve the reliability and robustness [14,19,20,28,29,30]. However, using helper data may leak some information about the secret keys, and ECC circuits cause significant hardware and software overheads. Temporal majority voting (TMV) is one of the simplest ECCs, which is a repetition code and is based on sampling PUF cells multiple times and selecting the majority sample. Bose Chaudhuri Hocquenghem (BCH) codes are another popular ECC that are usually utilized as a final error-correction technique. It is inefficient to use BCH codes alone when the bit-error rate (BER) of native responses is high. Thus, some additional stages are required to be applied to raw PUF responses before using ECC mechanisms due to varying characteristics and unpredictable behaviors of DRAM cells in different measurements under normal/unstable ambient conditions.

As mentioned above, current DRAM PUFs, including latency-based ones, mask unstable and unsuitable cells using filtering processes or selection algorithms [12,13]. However, these solutions limit the CRP space by disqualifying the unacceptable cells and cause extra time and implementation overheads. Additionally, they need specific algorithms to determine the accurate location (address information) of selected cells to indicate them in the challenges [31,32].

### 2.4. Motivation

As stated in Section 2.2, the current solutions of error-reduction in DRAM PUFs cause additional costs and cannot be efficiently implemented, particularly when the PUF is embedded in a resource-restricted IoT device. Adding considerable hardware overheads and increasing the implementation complexity on the device side, as well as limiting the CRP space of DRAM PUF, are some of the most important disadvantages of existing error-reduction methods and the key motivations of this work. Last but not least, it has been shown that most of error-reduction schemes such as fuzzy extractors have their own security defects [14]. The deep PUF mechanism eliminates the need for error-reduction strategies and their overheads using a two-stage deep CNN-based mechanism for a lightweight authentication.

## 3. Proposed Deep PUF

Our proposed method substantially focuses on an authentication technique which is suitable for device identification with no extra overheads for resource-constraint nodes in an IoT network. In this work, we first generate DRAM PUF responses for different challenges over multiple measurements under various temperature conditions. We create a CRP database including each challenge with its corresponding responses (Section 4). Next, we take the advantages of CNNs to extract the shared features of generated responses as well as failure patterns. In such a way, the developed CNN learns the shared features of several responses generated for each challenge. Then, it will be able to recognize corresponding responses produced in all operating conditions. This method will help us to address the error-reduction issues and confront reliability requirements to organize an authentication technique. Deep PUF can be used as a standalone security mechanism or as a part of multi-factor authentication (MFA). The proposed authentication process is generally divided into two major stages: (i) an enrollment phase and (ii) an authentication phase.

### 3.1. Enrollment

**Figure 3:**
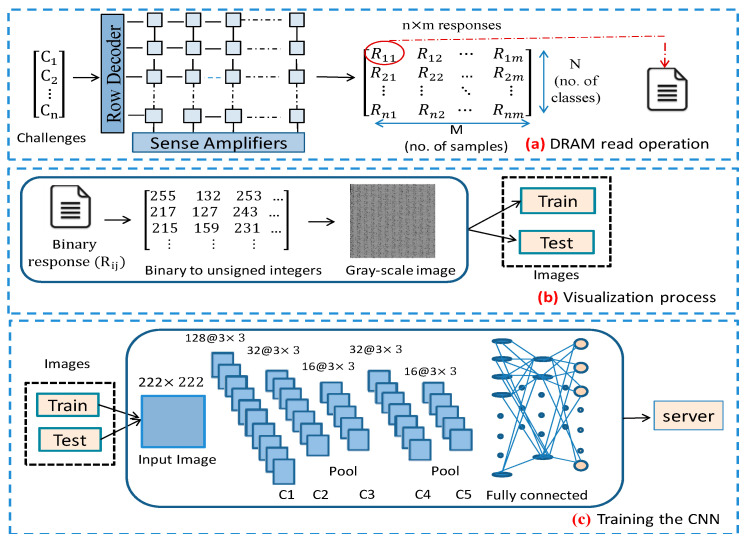
Enrollment Phase.

Figure 3 illustrates the entire enrollment procedure. It is made up of three steps: reading the DRAM PUF responses, converting them to gray-scale images and training the CNN.

In the first stage, the characterization of DRAM responses based on a particular DRAM PUF technology (e.g., t_RCD_ based, t_RP_ based, etc.) over multiple iterations and under various ambient conditions is analyzed. Then, considering the necessary features to develop a successful classifier, challenges are selected, which contain the address of memory blocks and the input data patterns. The output responses as well as failure bits for each challenge are categorized without any modification (see Figure 3a). The number of measurements to obtain the comprehensive features of whole possible responses for each challenge can be effectively set based on intrinsic robustness evaluation.

To organize the training dataset for CNN, we transform binary responses into two-dimensional arrays of unsigned integers and finally gray-scale images via the visualization phase (see Figure 3b). Therefore, the required dataset is constructed during the first and second steps. After creating the dataset of the PUF device, it is necessary to extract the chief and common features of the samples (responses) of each class (challenge) by training a CNN. The CNN developing procedure, including the hyper-parameter settings, should be optimized in consideration of PUF characteristics. The most important and influential factors of PUF during CNN training and performance optimization (classification accuracy) are robustness and uniqueness.

- Robustness: determines the effects of different operating conditions on output responses. This property affects the similarity of samples in a single class and accuracy of classification results. Robustness of DRAM PUF can be calculated using intra-Hamming distance (HD) or intra-Jaccard index values.
- Uniqueness: enough difference between two responses using two distinct DRAM blocks results in uniqueness. This factor shows the difference of samples belonging to separate classes and can be determined by computing inter-class HD. Figure 3c depicts the developed deep CNN which is trained on the generated dataset and learns the failures behavior under various measurements.

We recognize two major variables which significantly affect the classification accuracy and finally the authentication performance.

- Stability of operating conditions: locating the PUF device in a stable ambiance in which the variety of conditions (e.g., temperature, voltage) is not appreciable, causes more consistency inside each class and results in better accuracy. Due to the PUF sensitivity to environmental conditions, in an environment with varying temperatures, the number of bit failures in each measurement and the way the failures are distributed may cause samples to be far different than usual. In this case, deep PUF requires involving the responses of all possible temperatures to extract entire failure features, thereby leading an accurate classification.
- Variety of blocks and input patterns: one scenario is to organize the classes using only a single memory block and writing different patterns into it as the challenges, and the other one is exploiting various blocks. If only one memory block is utilized to perform the PUF, it is necessary to provide the challenges based on different input data patterns. However, in the case of using multiple blocks, the challenges can be configured by the same data for all blocks.

### 3.2. Authentication Phase

**Figure 4:**
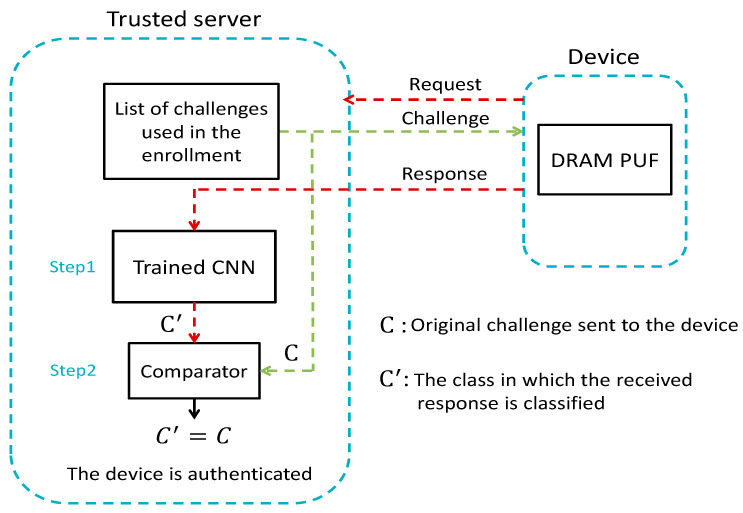
The authentication phase includes communication process and verification steps in the server.

Figure 4 shows the authentication procedure as well as the major parts of server and device. In each authentication request from the PUF device, the server sends one of the challenges (prearranged class labels) to the device to configure a particular DRAM block as a PUF. Next, the device generates the corresponding response and sends it to the trusted server. Then, the server authenticates the device in two steps:

1. The received raw bits are classified using CNN, structured during the enrollment phase.
2. The detected label is compared with the original challenge.

The device will be authenticated if the class label in which the response was categorized matches the original challenge. Otherwise, if the class of received response and the sent challenge are different, the authentication will be discarded, and the server will reject the device’s request to exchange data. In this structure the burden of device authentication is completely moved to the server, which has almost no resource limitations. Therefore, deep PUF enables an authentication process for resource-constrained nodes without any extra implementation overheads.

## 4. DRAM Experiments and Observations

**Figure 5:**
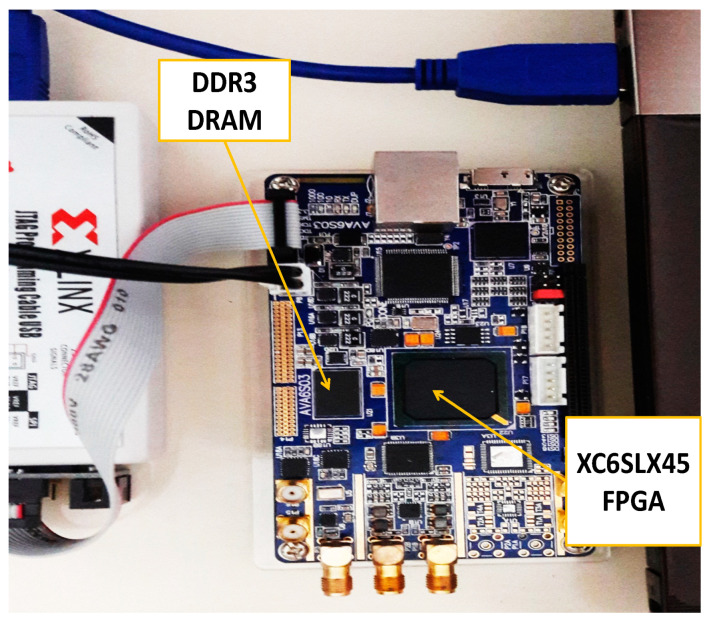
Experimental setup.

In this section, we present DRAM PUF implementation results and check the behavior of responses that are generated using latency-based technologies (i.e., t_RCD_ and t_RP_ PUFs). The experimental evaluations are conducted using a DDR3 DRAM module. Figure 5 shows our experimental setup. We examine the characteristics of both latency PUFs to make a better decision considering the CNN requirements. We read DRAM values in different conditions to evaluate the robustness and uniqueness of DRAM blocks.

**Figure 6:**
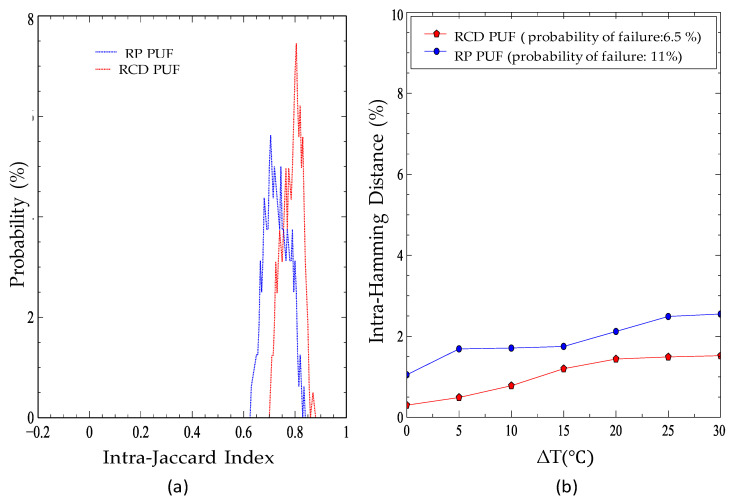
(**a**) Distributions of intra-Jaccard indices calculated between responses for t_RCD_ and t_RP_ PUFs. (**b**) The average intra-Hamming distance (HD) for t_RCD_ and t_RP_ PUFs with different temperatures (reference temperature is 25 °C).

Table 1 shows the parameter values of our experiments that are the same for both evaluated structures. To measure the robustness of each PUF, we extract multiple responses over several iterations at varying temperatures (25–55 °C). Figure 6a shows the intra-Jaccard of PUF responses using both t_RCD_ and t_RP_ reduction-based methods. The intra-Jaccard index determines the similarity of two PUF responses for the same challenge. This is calculated as $\frac{R_{1}\cap^{\;}{\;R}_{2}}{R_{1}\cup^{\;}{\;R}_{2}}$ for two sets of responses, where $R_{1}\cap^{\;}R_{2}$ indicates the size of the shared failures and the $R_{1}\cup^{\;}R_{2}$ is the total number of failures in $R_{1}$ and $R_{2}$. A Jacard index close to 1 shows the more similarity between $R_{1}$ and $R_{2}$. In this work, this metric is used to check the repeatability and robustness of DRAM PUF responses. These results are based on the average values that we have gathered by checking multiple samples at each temperature. We also have tested the sensitivity of PUF responses to temperature variations by intra-HD calculations; the results are shown in Figure 6b, indicating the reliability of DRAM PUF responses and also the similarity of samples creating a class. Another principal factor affecting the performance of deep PUF is uniqueness, which measures the difference between failure distributions into two different memory blocks. We have analyzed this factor by comparing multiple samples belonging to various blocks of the DRAM module using inter-HD. Table 2 presents the average uniqueness for t_RCD_ and t_RP_-based methods considering the average number of bit failures in each block.

After analyzing the robustness and uniqueness for both t_RCD_ and t_RP_ PUFs, we realize that they have desirable characteristics to develop a classifier and organize deep PUF. These characteristics include the similarity among the samples into each class and variety among samples from different classes. Table 3 summarizes the generic HD values for stable and unstable conditions, which can be two possible scenarios during a deep PUF configuration. We focus on the t_RCD_-based PUF that comparatively has more intra-class consistency.

## 5. Development of CNN Model

### 5.1. Dataset Creation

Each DRAM PUF is configured by sending M challenges to the device and generating N responses for each of them. The CNN is trained on and organized to classify these challenges from N × M responses. Each challenge is defined as a class label, and the corresponding responses are the class samples. The value of N can be adjusted by the total number of classes and the environmental conditions, which significantly determine the consistency of samples in each class. In order to examine the effect of important variables, including stability of ambient conditions and variety of input patterns (see Section 3), we generate four datasets considering the following scenarios:

1. The same input pattern (all “1”s) is used to characterize all blocks and the operating conditions are stable (room temperature and nominal voltage).
2. Different input patterns (0x00, 0x01… 0xFF) are used for different blocks and the conditions are stable.
3. The same input pattern is used to characterize all blocks and the operating conditions are unstable.
4. Different input patterns are used for different blocks and the conditions are unstable.

The inputs of the network are visualized DRAM data, which are converted to gray-scale images, as demonstrated in Section 3. The samples of different classes are randomly shuffled and each dataset is divided into 80% training, 20% testing data. Table 4 includes the main features of generated datasets.

### 5.2. Training the Classifier

The proposed deep PUF consists of convolution, max-pooling and fully connected layers. The summary of our network model is presented in Table 5. The activation function of all layers except the output layer is the rectified linear unit (ReLU). Classification is performed by determining the probability of different classes using the Softmax. In this classifier, we utilize categorical cross-entropy as the loss function and the Adam algorithm as the optimizer. Algorithm 1 shows the proposed scheme in form of a pseudo-code containing dataset generation, training process and testing process.
**Algorithm 1** Convolutional neural network (CNN)-based classification**Dataset generation**Input: a set of challenges, including the address of PUF segment and input pattern: ($C_{1},C_{2},\ldots ,{\;C}_{N}$)Output: collections of images corresponding to different challenges: ($S_{1},{\;S}_{2},\;\ldots ,{\;S}_{N}$)Process:   // build N folders: folder_1, folder_2,…, folder_Nfor i=1 to N do   //N: number of classes      for k=1 to M do   // M: number of measurements $\;\;\;\;\;\;\;\;\;\;\mathrm{Write}\;\mathrm{the}\;\mathrm{input}-{\mathrm{pattern}\;\mathrm{in}\;\mathrm{the}\;\mathrm{address}\;\mathrm{using}\;C}_{i}\;;$$\;\;\;\;\;\;\;\;\;\;\mathrm{Change}\;\mathrm{the}\;\mathrm{timing}\;\mathrm{parameter}\;\left(t_{\mathrm{RCD}}\right);\;$$\;\;\;\;\;\;\;\;\;\;\mathrm{Read}\;\mathrm{the}\;\mathrm{DRAM}\;\mathrm{segment}\;\to {\;R}_{k}\;;$$\;\;\;\;\;\;\;\;\;\;\mathrm{Visualize}\left(R_{k}\right)$    //Convert the $R_{k}$ to integer values and a gray-scale image ${\;\;\;\;\;\;\;\;\;\;\mathrm{Store}\;R}_{1},{\;R}_{2},\ldots ,{\;R}_{M}\;\mathrm{into}\;\mathrm{the}\;\mathrm{folder}\_i\;;$     end forend forOutput: a dataset including N folders with M images in each folder.
**Training process**Input: a collection of labeled images (responses): $\left(s_{1},s_{2},\ldots \;,s_{M},{\;s}_{M+1},\ldots ,{\;s}_{\mathrm{MN}}\right)$
Output: a collection of features assigned to different labelsProcess: for k=1 to e do // e: number of epochs     for i=1 to M×N do   // N: number of classes, M: number of samples for each class$\;\;\;\;\;\;\;\;\;\;\mathrm{Get}\;\mathrm{the}\;\mathrm{sample}\;\mathrm{with}\;\mathrm{the}\;\mathrm{label}\;\to \left({\;s}_{i}\;,{\;y}_{i}\right);$$\;\;\;\;\;\;\;\;\;\;\mathrm{Gain}\;\mathrm{the}\;\mathrm{features}\;\to {\;f}_{i};\;$     // after applying the defined layers$\;\;\;\;\;\;\;\;\;\;\mathrm{Assign}\;\mathrm{the}\;\mathrm{features}\;\mathrm{to}\;\mathrm{the}\;\mathrm{label}\;\left(f_{i}\;,{\;y}_{i}\right);$     end for     for j=1 to N do          Build collection of features for each class→ ($F_{j}$,$Y_{j}$);     end for$\;\;\;\;\;\mathrm{Build}\;F=\left(F_{1},{\;F}_{2},\;\ldots ,{\;F}_{N}\right)$     Update the features → F; end forBuild the final collection of features Output: $\left(F_{1},{\;Y}_{1}\right),\;\left(F_{2},{\;Y}_{2}\right),\;\ldots ,\;\left(F_{N}\;,{\;Y}_{N}\right)$
**Testing process**Input: x   // testing sampleProcess: apply the CNN $\mathrm{Select}\;\left(x^{\prime },y^{\prime }\right),\;p\left(x^{\prime },y^{\prime }\right)=\max\left\{p1\left(x^{\prime },\;y1\right),\;p2\left(x^{\prime },y2\right),\;\ldots ,\;\mathrm{pN}\left(x,\;\mathrm{yN}\right)\right\}$   // p: the probability vector calculated by SoftmaxfunctionAssign y′ to the x

### 5.3. Performance Metrics

The proposed network is simulated using the Keras library of Python and TensorFlow backend. In this work, we analyze CNN performance considering two major variables described in Section 3, using datasets based on four presented scenarios (see Section 5.1). Table 6 shows the accuracy results of training the network. The influence of using the data augmentation technique is examined and is presented in Table 6. This technique improves the accuracy of classification by expanding the training dataset.

In the worst scenario (unstable environments and the same inputs), the accuracy of classification is 92.29%, which can achieve 97.79% in the case of applying different input patterns in a stable condition.

**Figure 7:**
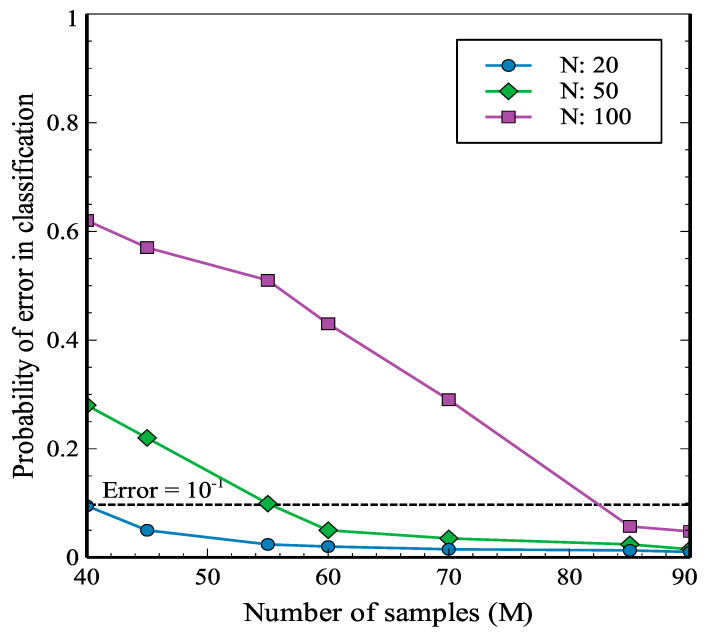
Probability of error in classification as a function of the number of measurements for the different number of classes (N = 20, 50 and 100).

Additionally, for a classification problem with N challenges, the number of samples for each label (M) is an influential parameter to achieve better accuracy. In this work, the authors have accomplished the classification process using 90 samples for each class, which is a reasonable decision for a PUF-based mechanism leading to a cost-effective enrollment procedure. However, it is functional to add more samples to each class in order to generate a more comprehensive dataset and achieve satisfactory accuracy depending on the application. Figure 7 illustrates the average accuracy of classification after 60-epoch training as a function of the number of samples in each class, considering the different number of classes. The experiment is performed by writing the same input pattern into various memory blocks at room temperature. The results indicate that it is feasible to achieve an error less than 10^−1^ and even near 10^−2^ by adjusting the number of measurements during the enrollment phase.

## 6. Security Analysis and Discussion

### 6.1. Security and Robustness

**Figure 8:**
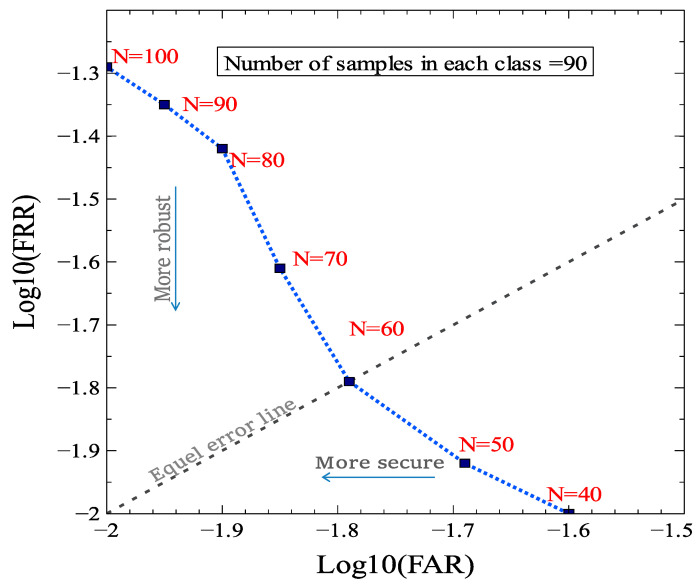
The values for false acceptance rate (FAR) and false rejection rate (FRR) for deep PUF.

The security and robustness of authentication mechanisms are generally measured using two popular metrics: the false acceptance rate (FAR) and the false rejection rate (FRR). Generally, these two undesirable errors are defined considering the major PUF properties (intra-HD and inter-HD), and there is a tradeoff between them which can be controlled by a threshold for obtaining a suitable FAR and FRR [33]. The threshold is determined by effective parameters depending on the application. In this paper, FAR refers to the probability that a wrong response is verified as the true response from the target device and FRR is the probability of wrongly rejecting the target entity’s response. Based on the proposed authentication mechanism, the classification of received response is a major stage in accepting or rejecting it. When the server sends a challenge to the target device, the probability that the corresponding response is rejected directly depends on the result of classification of the response, which is compared to the original class in the next stage. Thus, the accuracy of classification significantly affects the FRR, and the probability of misclassification determines the FRR. However, the FAR value is not directly influenced by the rate of classification error and depends on CNN features. Assuming that a wrong response from an invalid device is received, it can be classified in each class with the same probability due to the uniqueness of PUF responses generated by different DRAM blocks. Therefore, the FAR is about 1/N, where N indicates the number of classes of the trained CNN. In Figure 8, the values for the FAR and FRR for deep PUF are shown and compared to some other PUFs. The threshold used for controlling the tradeoff between FAR and FRR can be determined by the number of classes. With a smaller number of classes (N = 60), the FAR and FRR achieve an equal value (0.016), but as N increases, the FAR and the accuracy of classification decrease leading to a higher FRR. However, the dependence of accuracy on other features (e.g., number of samples in each class and CNN model) can address this issue and enable a desirable FRR. In such a scenario, it will be possible to minimize both the FAR and FRR considering the major features, such as increasing the accuracy and the number of CNN classes simultaneously.

More generally, when a device is being authenticated, the probability of error during the response verification is influenced by both inter-device HD and inter-class HD. in Deep PUF, generating enough samples of each class using different ambient conditions, provides more involvement and minimizes the FRR. However, in other mechanisms, the response space is not large enough, and it is difficult to control both these errors.

### 6.2. Performance Comparisons

Deep PUF employs the t_RCD_-based PUF mechanism to generate raw DRAM data, proposed in [12]. This method uses a filtering procedure to extract the reliable cells and form the output response, which significantly increases the evaluation period. Deep PUF enables a lower evaluation time than t_RCD_-based PUF technology due to removing the filtering mechanism. The evaluation period of deep PUF can be measured in a way similar to t_RCD_-based PUFs, expressed by Equation (1).

(1) T=[(block_size)/(number of bytes in each read)]×(time needed for each read)

We also experimentally measure the evaluation time of deep PUF to confirm Equation (1). The average result of multiple evaluations is 0.95ms, which is almost equal to the value calculated by Equation (1). This period is much lower than t_RCD_-based PUF’s evaluation time, which is 88.2ms. Note that the evaluation time has been measured for the PUF operation on the device and does not include the time of authentication process on the server side. Furthermore, t_RCD_-based PUF needs at least two DRAM ranks: one for PUF operation and one for counting the latency failures. The proposed deep PUF is operational with only one rank and is appropriate for low-cost systems. Additionally, both t_RCD_-based [12] and t_RP_-based PUFs [13] require post-processing error correction algorithms that cause significant time and hardware overheads. On the other hand, retention-based PUFs [12,28] require a long period of time (order of minutes) to extract sufficient failure bits and generate reliable signatures, which makes the DRAM rank unavailable for a long time.

### 6.3. Security Discussion and Countermeasures against Possible Attacks

In this authentication structure, the server only stores the list of original challenges and the information of trained CNN. Therefore, the responses are not stored in the server storage. This property improves the security against the insider attack that can be executed by a malicious entity with authorized server data access.

Snooping-based and modeling attacks take place when multiple CRPs related to the same memory block are accessed and learned by the adversary [34]. These attacks can be prevented by exploiting various and separate blocks of the DRAM during the deep PUF configuration. Since, as the implementation results indicate, different memory blocks entail various features, it is difficult to model all blocks of a DRAM chip using the limited leaked CRPs.

PUF re-use attacks are theoretically possible in the deep PUF-based authentication mechanism, as the server may send identical challenge to a PUF device. In such a way, if the adversary can intercept in the process, the authentication system can suffer from re-use attacks. One alternative way to avoid such attacks is to utilize a simple encryption algorithm to enhance the security of transmitted data [18]. One-time-use protocol is another way, which can be employed to prevent re-use attacks considering the application requirements [35]. Another solution is to include erasability and certifiability as two additional features in PUF application [36]. The property of certifiabilty provides an offline certification to check and verify the expected features from the PUF responses. Erasability can be conducted using reconfiguration methods, which are compatible with DRAM PUF organization. We leave the implementation and validation of these techniques to future work.

## 7. Conclusion and Future Work

In this paper, we present a new DRAM PUF-based authentication method that exploits a deep CNN to configure a strong PUF with an expanded CRP space and light-weight implementation. This method eliminates additional error-correction mechanisms and their overheads. The experimental analysis of DRAM latency-based PUFs with their characterizations under various conditions and the feasibility of developing a precise CNN are elaborated in this paper. We organize a CNN-based classifier using real DRAM data. We examine the effects of major parameters by generating four datasets based on four key scenarios and applying them to CNN. Based on simulation results, we show that in the case of using various input patterns for different blocks at varying ambient conditions, the proposed classifier can achieve 94.9% accuracy. We also propose a two-step authentication technique, including response classification and label verification. This method can noticeably minimize identification errors and leads to higher reliability than classification accuracy. The proposed scheme can be employed as a stand-alone mechanism or as a part of multi-factor authentication. Additionally, our proposed deep PUF moves all implementation overheads to the server and is appropriate for low-cost and resource-constrained devices. Finally, we demonstrate that deep PUF significantly reduces the evaluation time and hardware overheads compared with the existing DRAM PUFs. One direction for the future work may be derived from studying how to extend the proposed approach to other strong PUFs and analyzing existing techniques to protect deep PUF against possible attacks to achieve a secure and light-weight authentication protocol.

## Figures and Tables

**Table 1:** Experimental parameters used for t_RCD_ and t_RP_ reduction-based DRAM PUFs.

Reduced time	5 ns
Block size	200 Kb
Input pattern	All “1” s
Block address	Various
Number of tested blocks	200

**Table 2:** Average inter-HD for t_RCD_ and t_RP_ PUFs.

Mechanism	Average Inter-HD	Average Probability of Failure (for 200 Blocks)
RCD PUF	12.15%	6.5%
RP PUF	20.21%	11%

**Table 3:** Inter-class (HD) and intra-class HD, considering environmental conditions.

Mechanism	Stable Environmental Conditions		Unstable Environmental Conditions	
	Intra-Class HD	Inter-Class HD	Intra-Class HD	Inter-Class HD
RCD PUF	0.32%	12.7%	1.44%	11.64%
RP PUF	1.05%	21.1%	3.41%	19.33%

**Table 4:** The specifics about the generated datasets.

Total number of images in each dataset	9000
Number of classes (challenges)	100
Number of samples (responses)	90
Tested temperatures	25–55 °C
Percentage of training data	80
Percentage of test data	20
The resolution of images (pixels)	222 × 222

**Table 5:** Architecture of the convolutional neural network (CNN) used for classification.

Layer	Dimension
Convolution 2D	(222, 222, 128)
Convolution 2D	(220, 220, 32)
Max pooling	(109, 109, 32)
Convolution 2D	(107, 107, 16)
Convolution 2D	(105, 105, 32)
Max pooling	(52, 52, 32)
Convolution 2D	(50, 50, 16)
Max pooling	(24, 24, 16)
Flatten	9216
Dense	145
Dense	75
Dense	Number of classes

**Table 6:** Accuracy of classification considering different scenarios (N = 100, M = 90).

Experimental Conditions	Accuracy of Classification (%)			
	Same Input Pattern for All Blocks		Different Input Patterns	
	Augmented Data	Original Data	Augmented Data	Original Data
Stable temperature	96.12	94.66	97.79	97.15
Various temperatures	92.29	91.03	94.9	94.33

## Data Availability

Not applicable.
